# Respiratory infections drive hepcidin-mediated blockade of iron absorption leading to iron deficiency anemia in African children

**DOI:** 10.1126/sciadv.aav9020

**Published:** 2019-03-27

**Authors:** Andrew M. Prentice, Amat Bah, Momodou W. Jallow, Amadou T. Jallow, Saikou Sanyang, Ebrima A. Sise, Kabiru Ceesay, Ebrima Danso, Andrew E. Armitage, Sant-Rayn Pasricha, Hal Drakesmith, Miriam Wathuo, Noah Kessler, Carla Cerami, Rita Wegmüller

**Affiliations:** 1 MRC Unit The Gambia at LSHTM, Atlantic Road, Fajara, The Gambia.; 2 MRC Human Immunology Unit, MRC Weatherall Institute of Molecular Medicine, University of Oxford, John Radcliffe Hospital, Oxford, UK.; 3 Walter and Eliza Hall Institute for Medical Research, 1G Royal Parade, Melbourne, Parkville, Victoria 3052, Australia.; 4 Department of Genetics, University of Cambridge, Cambridge, UK.; 5 GroundWork, 7306 Fläsch, Switzerland.; 6 Human Nutrition Laboratory, Institute of Food, Nutrition and Health, Department of Health Sciences and Technology, ETH Zurich, Zurich, Switzerland.

## Abstract

Iron deficiency anemia (IDA) is the most prevalent nutritional condition worldwide. We studied the contribution of hepcidin-mediated iron blockade to IDA in African children. We measured hepcidin and hemoglobin weekly, and hematological, inflammatory, and iron biomarkers at baseline, 7 weeks, and 12 weeks in 407 anemic (hemoglobin < 11 g/dl), otherwise healthy Gambian children (6 to 27 months). Each child maintained remarkably constant hepcidin levels (*P* < 0.0001 for between-child variance), with half consistently maintaining levels that indicate physiological blockade of iron absorption. Hepcidin was strongly predicted by nurse-ascribed adverse events with dominant signals from respiratory infections and fevers (all *P* < 0.0001). Diarrhea and fecal calprotectin were not associated with hepcidin. In multivariate analysis, C-reactive protein was the dominant predictor of hepcidin and contributed to iron blockade even at very low levels. We conclude that even low-grade inflammation, especially associated with respiratory infections, contributes to IDA in African children.

## Teaser

Inflammation from respiratory infections contributes to iron deficiency anemia in children by blocking iron absorption.

## INTRODUCTION

Iron deficiency (ID) and iron deficiency anemia (IDA) are highly prevalent in low-income countries (*1*). The 2017 Global Burden of Disease exercise estimated that 1.224 billion people suffer from IDA (*2*). In most of sub-Saharan Africa and many South Asian countries, it is the leading cause of years lived with disability and, by this measure, outweighs all other nutritional deficiencies, hemoglobinopathies, and hemolytic anemias put together (*2*). The etiology of ID and IDA has hitherto been ascribed to poor diets lacking in animal products and containing dietary phytates and related anti-nutrients and to infections (especially helminths and malaria). Rigorously implemented efficacy trials with iron supplementation accompanied by multiple micronutrients achieve some resolution of ID and anemia, but the meta-analyzed results show a benefit of only 3 to 5 g/liter increase in hemoglobin (Hb) (*3*–*6*).

Hepcidin is the master regulator of iron metabolism, and its discovery offers fresh insights into the pathophysiology of ID and its consequent anemia. Hepcidin has evolved to regulate duodenal iron absorption and tissue iron distribution in response to competing signals relating to iron status and infection/inflammation (*7*, *8*). Hepcidin suppression, driven by ID or a high erythropoietic drive, facilitates iron absorption and distribution. Hepcidin expression, driven by iron sufficiency/overload, infection, or inflammation, blocks iron absorption and sequesters iron in macrophages (*7*, *8*). Hepcidin up-regulation in the acute-phase response drives the plasma hypoferremia that restricts iron availability to potentially pathogenic microorganisms (*9*).

Previous studies in African children have shown that hepcidin levels are variably influenced by the combined effects of malaria, iron status, and inflammation (*10*, *11*) and can reliably identify IDA and distinguish it from the anemia of infection (*12*). The objective of the current study was to examine the frequency and duration of elevated hepcidin to levels that would inhibit iron absorption and to describe the associations between elevated hepcidin, inflammation, infections, and iron status. To achieve this aim, we analyzed weekly plasma hepcidin levels over 12 weeks in a large cohort of young Gambian children aged 6 to 23 months participating in a randomized controlled trial (*13*).

## RESULTS

### Children maintain consistent levels of hepcidin

**Fig. 1:**
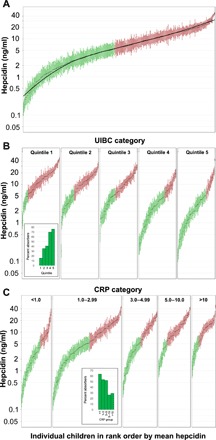
Gambian children maintain highly characteristic hepcidin levels over 12 weeks of study. (**A**) Twelve-week geometric mean hepcidin values (±SE) for individual children put in rank order from lowest to highest. Individual means and the smoothed curve for all children are shown in black. Data illustrated are for all 388 children with more than six serial measurements. Green indicates habitual iron absorbers (hepcidin <5.5 ng/ml); red indicates habitual iron blockers (hepcidin >5.5 ng/ml). (**B**) Data from (A) are recategorized according to quintiles of UIBC. UIBC concentrations: Q1, 33.7 to 52.4 μM; Q2, 52.4 to 59.3 μM; Q3, 59.3 to 66.8 μM; Q4, 66.8 to 74.3 μM; Q5, 74.3 to 120 μM. Green indicates habitual iron absorbers (hepcidin <5.5 ng/ml); red indicates habitual iron blockers (hepcidin >5.5 ng/ml). The proportion of iron absorbers in each quintile is indicated in the inset panel and was as follows: Q1, 15.6%; Q2, 35.5%; Q3, 40.8%; Q4, 69.7%; Q5, 76.0%. (**C**) Data from (A) are recategorized according to clinical categories of CRP (mg/liter). Green indicates habitual iron absorbers (hepcidin <5.5 ng/ml); red indicates habitual iron blockers (hepcidin >5.5 ng/ml). The proportion of iron absorbers in each group is indicated in the inset panel: CRP <1, 63.3%; CRP 1 to 3, 53.6%; CRP 3 to 5, 52.2%; CRP 5 to 10, 26.5%; CRP >10, 29.2%.

The availability of serial measurements allowed us to examine the variability of plasma hepcidin levels across the 12 weeks of study. Table 1 summarizes the baseline characteristics. The 407 participating subjects were selected as being anemic at recruitment (Hb < 11 g/dl) and made up 75% of the potentially eligible children. The proportion iron deficient at baseline was 58.5% [defined at ferritin <15 μg/liter if C-reactive protein (CRP) <5 mg/liter or ferritin <30 μg/liter if CRP >5 mg/liter]. Estimates based on raised soluble transferrin receptor (sTfR) (45.7%) or low hepcidin (49.1%) were similar, given the uncertainties of how best to define ID in young children. Using the lower limit of normal for mean corpuscular volume (MCV) in African Americans of 63 fl, 52.6% had IDA. Hepcidin increased gradually in response to the universal iron supplementation in group 1 (by 0.058 ng/ml per day; *P* < 0.0001) but not in the two screen-and-treat groups. To account for this serial increase, we incorporated “visit day” into all analyses. Figure 1A shows the mean ± SE of log hepcidin for each child displayed in rank order for all 388 children who had six or more hepcidin measurements. It displays a remarkable constancy of hepcidin with a highly significant *F* ratio for the between-child variability versus within-child variability (*F* = 8.2, df = 386, *P* < 0.0001). Twelve-week mean hepcidin levels ranged from 0.18 to >40 ng/ml. Note that the smaller SEs among the children with the highest hepcidin levels are caused by allocating the value of 50 ng/ml whenever enzyme-linked immunosorbent assay (ELISA) readings were beyond the calibration curve, and there was insufficient sample to dilute and repeat the analysis. Their true mean values and associated variance would have been higher. Fifty-two percent of the children (those indicated in red in Fig. 1A) consistently maintained hepcidin levels above the threshold of 5.5 ng/ml, which predicts that they were blocking iron absorption for the greater part of the 12 weeks studied.

**Table 1:** Baseline characteristics of subjects. *N* = 407 (209 girls, 198 boys). WBC, white blood cells.

	**Mean**	**5th and 95th** **percentiles**
Age at enrolment (months)	15.4	7.7, 22.2
Length-for-age (*z* score)	−1.00	−2.45, 0.51
Weight-for-age (*z* score)	−1.19	−2.67, 0.39
Weight-for-height (*z* score)	−0.90	−2.36, 0.54
Hepcidin (ng/ml)*	3.09	0.05, 39.05
Hb (g/dl)	9.78	7.30, 11.90
Hematocrit (%)	27.9	21.8, 34.1
MCV (fl)	62.8	50.2, 74.1
MCHC (g/dl)	35.1	32.7, 37.3
WBC (×10^9^ per liter)	11.9	6.2, 19.7
Granulocytes (×10^9^ per liter)	4.53	2.1, 8.0
Lymphocytes (×10^9^ per liter)	6.32	3.17, 10.53
Ferritin (μg/liter)*	8.18	0.1, 62.2
UIBC (μM)	64.5	40.9, 92.8
TSAT (%)	7.87	0.80, 20.9
sTfR (mg/liter)	8.92	4.73, 15.06
Serum iron (μM)	5.70	0.7, 13.4
CRP (mg/liter)*	2.30	0.1, 24.5
AGP (g/liter)*	1.18	0.64, 2.23
ID		
Ferritin <15 μg/liter^†^	50.9%	
Ferritin <15 μg/liter if CRP <5 mg/liter or ferritin <30 μg/liter if CRP >5 mg/liter	58.5%	
sTfR >8.3 mg/liter	45.7%	
Hepcidin <5.5 ng/ml	49.1%	
IDA		
MCV <63 fl^‡^	52.6	
MCV <71 fl^§^	87.0	

*Geometric means.

†Defined as ferritin <15 μg/liter.

‡Defined as MCV <63 fl (lower limit for African Americans).

§Defined as MCV <71 fl (lower limit for Caucasian Americans).

### Influence of iron status on hepcidin

Univariate analysis of factors predicting hepcidin revealed the associations listed in Table 2. None of a child’s sex, age, or anthropometry were associated with their hepcidin level, nor were Hb or hematocrit. Long-acting measures of positive iron status [ferritin, low unsaturated iron binding capacity (UIBC), low transferrin, low sTfR, high MCV, and mean corpuscular hemoglobin (MCH)] were all strongly inversely associated with hepcidin (*P* < 0.0001). Serum iron and transferrin saturation (TSAT), which respond rapidly to recent iron intake, were not associated with hepcidin. Surprisingly, whether the children received iron supplements over the previous week also was not associated with hepcidin. Ferritin is both a marker of iron status and inflammation, and therefore, it is not an optimal marker of iron status. In this dataset, UIBC, which incorporates information from raised transferrin (an indicator of physiological effort to acquire more iron) and low serum iron, was the best iron status predictor of hepcidin (note that sTfR was also a good predictor of hepcidin, but UIBC outperformed sTfR in the multivariate analysis described below).

**Table 2:** Factors associated with children’s characteristic hepcidin levels. The multivariate analysis includes all the variables with a significant influence based on forward selection with *P* < 0.05. Prior iron, iron supplementation over the previous 7 days; NS, not significant.

**Predictor**	**Positive/negative** **association**	*F* ratio*	***P***	***R*^2^**
Univariate analysis				
Iron indices				
Log ferritin (also affected by inflammation)	Positive	37.0	<0.0001	17.1
Transferrin	Negative	15.2	<0.0001	13.4
UIBC	Negative	19.9	<0.0001	13.2
sTfR	Negative	22.0	<0.0001	13.0
Serum iron	–	4.4	0.04	–
TSAT	–	0.0	NS	–
Prior iron	–	0.1	NS	–
Hb	–	1.3	NS	–
MCV	Positive	15.2	<0.0001	8.1
MCH	Positive	21.0	<0.0001	11.4
Inflammatory markers				
Log CRP	Positive	111.5	<0.0001	14.2
Log AAGP	Positive	45.4	<0.0001	6.5
Total WBC	Positive	5.3	<0.02	1.7
Granulocytes	Positive	39.1	<0.0001	3.9
Lymphocytes	Positive	6.0	0.014	1.7
Granulocyte-to- lymphocyte ratio	Positive	48.2	<0.0001	
Fecal inflammatory marker				
Log calprotectin	–	0.03	NS	–
Multivariate analysis				
Log ferritin	Positive	10.0	0.0015	26.6
UIBC^†^	Negative	11.3	0.0013	
Log CRP	Positive	46.6	<0.0001	
Granulocyte-to-lymphocyte ratio	Positive	14.9	<0.0001	
Stratified analysis of CRP as predictor of hepcidin^‡^				
CRP range				
All (*n* = 370)	Positive	111.5	<0.0001	14.2
<10 (*n* = 322)	Positive	65.2	<0.0001	10.9
<5 (*n* = 258)	Positive	35.6	<0.0001	8.9
<3 (*n* = 189)	Positive	23.9	<0.0001	7.2
<1 (*n* = 60)	Positive	14.4	0.0002	3.6

*Adjusting for individual subject (discrete variable) and week (continuous variable). The *F* ratios and *P* values shown are from the same multivariate model, including the four variables shown as well as subject and week.

†UIBC and sTfR were mutually replaceable in the multivariate analysis.

‡Log hepcidin against log CRP with adjustment for individual subject (discrete variable) and study week (continuous variable).

Figure 1B illustrates children’s characteristic mean hepcidin values for each quintile of UIBC. As expected, the mean plasma hepcidin decreased across the quintiles and the proportion of iron absorbers increased from 16 to 76% (see inset panel).

### Influence of inflammation on hepcidin

The markers of inflammation [CRP and α1-acid glycoprotein (AGP)], granulocyte number, and the granulocyte-to-lymphocyte ratio were strongly positively associated with hepcidin in univariate analysis (all *P* < 0.0001) (Table 2). Ferritin was also strongly positively associated but is a confounded index of both inflammation and iron status. Total white cells and lymphocytes were weaker but significant positive correlates. Notably, fecal calprotectin was not associated with plasma hepcidin.

Figure 1C shows that, after allocating children to the five clinically recognized CRP strata, there remained a very substantial interindividual difference across the subgroups. Mean plasma hepcidin increased with increasing CRP, and the proportion of iron absorbers decreased from 63 to 29% (see inset panel). CRP remained significantly associated with plasma hepcidin levels even among children with CRP <1 mg/liter, which is usually considered to indicate an absence of inflammation (Table 2). Even in the 200 children (52% of sample) who had mean CRP less than 3 mg/liter, indicating low levels of inflammation, 42% had a mean hepcidin above the threshold associated with iron blockade.

### Influence of infections

Maternal report of recent infection was associated with raised hepcidin but did not alter the proportion of iron absorbers versus nonabsorbers (Table 3). There were 425 registered adverse events (AEs), of which the subcategories fever, diarrhea, respiratory infections, and skin infections had sufficient events to warrant categorical analysis. Total AEs, fevers, and respiratory infections were strongly positively associated with hepcidin (all *P* < 0.0001); diarrhea and skin infections showed no detectable association.

**Table 3:** Multivariate analysis of maternal reports of infection and physician-notified AEs as a predictor of plasma hepcidin.

	**No. of events**	**Geometric mean hepcidin** **(% >5.5 ng/ml)**		***F* ratio**	P*
		**Illness** **Yes**	**Illness** **No**
Maternal reports^†^					
Any sickness	341	5.43 (45%)	4.33 (45%)	8	0.0052
Clinical records^‡^					
AEs	425	6.19 (59%)	4.23 (44%)	31	<0.0001
Fever	154	7.50 (68%)	4.30 (44%)	26	<0.0001
Diarrhea	129	4.95 (49%)	4.37 (45%)	1	NS
Respiratory	154	6.92 (64%)	4.31 (44%)	19	<0.0001
Skin	114	5.41 (54%)	4.36 (45%)	3	0.09

*P values obtained from multilevel linear modeling included subject, treatment group, and time as covariates.

†Number of reported illnesses at twice weekly fieldworker visits coinciding with hepcidin measurements (total *n* = 5113).

‡Number of recorded AEs coinciding with hepcidin measurements ±4 days (total *n* = 5113).

### Combined influences of iron status and inflammation on hepcidin

**Fig. 2:**
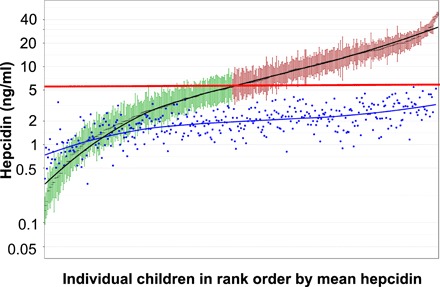
Attenuation in the range of interindividual characteristic hepcidin levels after multivariate adjustment for iron status and inflammation. Blue dots indicate each child’s estimated mean hepcidin value after multivariate adjustment for ferritin, CRP, UIBC, and granulocyte number. In the unadjusted data, green indicates habitual iron absorbers (hepcidin <5.5 ng/ml); red indicates habitual iron blockers (hepcidin >5.5 ng/ml). The horizontal red line shows the threshold separating absorbers from nonabsorbers.

Multivariate analysis including all variables associated with hepcidin that were significant in combination (UIBC, ferritin, CRP, and granulocytes) explained 26.6% of the variance in hepcidin. Figure 2 illustrates that the multivariable adjustment attenuated the range in mean hepcidin levels between children but, while highly attenuated, the between-child differences remained significant, though weakly so (*F* ratio = 1.8, df = 386, *P* < 0.001). Following this statistical elimination of the effect of inflammation (by use of the “estimated cell means” function in DataDesk, which predicts what the value would be in the absence of inflammation), all children had a mean hepcidin level below the threshold (5.5 ng/ml) and would hence be predicted to be good iron absorbers.

## DISCUSSION

Hepcidin, the master regulator of systemic iron metabolism, has evolved to exert exquisite control of iron intake and its distribution through various body compartments in response to the competing and opposing signals, indicating the body’s need for iron versus the threat of infection (*7*, *8*). In this study, the availability of weekly hepcidin measurements collected as part of a randomized trial (*13*) reveals several important new insights into the regulation of hepcidin and its role in mediating ID in rural African children. Although living conditions and infection profiles vary widely across poorer areas of low-income countries, our demonstration that respiratory infections seem to play a key etiological role in IDA could have important and widespread implications for community prevention programs.

We show that the children maintained remarkably consistent levels of hepcidin across the 12 weeks of study. Hepcidin has a half-life of <24 hours in adult humans (*8*) (likely shorter in young children) and is viewed as a relatively labile hormone with evidence for diurnal variation (*14*) and a modestly acute response to iron ingestion (*15*). The long-term stability of plasma levels in these children is therefore surprising, as is the very wide range, with some children maintaining exceptionally high hepcidin levels (>40 ng/ml averaged over 12 weeks). At the other extreme, there was a group of children maintaining hepcidin levels below our limit of detection, thus indicating complete hepcidin suppression.

Using stable isotope incorporation studies, we have previously determined that a hepcidin threshold (measured on the same ELISA) of 5.5 ng/ml effectively distinguishes iron absorbers from nonabsorbers (*12*, *16*). Over half of the children in this study maintained their mean 12-week hepcidin above this threshold and hence were physiologically inhibiting iron absorption despite the fact that all subjects were anemic at screening (Hb < 11 g/dl) and that, in spite of the iron supplementation, 61% of all ferritin levels throughout the study were below the accepted cutoffs, indicating ID in children (<15 μg/liter if CRP <5 mg/liter or <30 μg/liter if CRP >5 mg/liter) (*17*).

As anticipated, on the basis of the known physiology of hepcidin regulation and on previous studies, including in African children (*10*, *11*, *18*), hepcidin levels were strongly associated with measures of iron status. Somewhat surprisingly, it was the indices of longer-term iron status (ferritin, UIBC, sTfR, MCV, and MCH) that were good predictors and there was no discernible association with short-term indices (serum iron and TSAT) or with whether the children had received daily iron supplements for the preceding 7 days. In common with previous studies (*11*, *18*), Hb and MCH concentration (MCHC) were not associated with hepcidin levels. These results largely match previous data from a cross-sectional study of 6-month-old Kenyan infants by Jaeggi *et al*. (*10*), although they found a marginal association with Hb. Neither Jaeggi nor our study assessed liver iron levels, Hif-derived signals, or erythroferrone (*19*), all of which are additional regulators of hepcidin (*8*, *20*). Inclusion of these variables would likely have increased the proportion of total variance we could explain, and they remain unexplored components of hepcidin-mediated regulation of iron status in such populations. It is also likely that there are some genetic variants (e.g., in HFE, ferroportin, TMPRSS6, or others) that may contribute to the intersubject variance. The relatively normal distribution of log hepcidin makes it unlikely that there are strong effects of any monogenic variants; polygenic effects are more likely.

The fact that inflammation was strongly associated with raised hepcidin levels is also in line with the known influences on hepcidin expression. What is surprising is that CRP was the strongest correlate of hepcidin in these young children and was significantly associated even in the lowest category of CRP (<1 mg/liter), which is customarily assumed to indicate a lack of inflammation. Defining the source of this inflammation has major implications for the design of public health interventions to combat IDA. Surprisingly, we found only a weak association between maternal reports of sickness in their children and plasma hepcidin. The children in this study were free of infection at baseline (by protocol) and were mostly well for the duration of their follow-up; mothers reported illness on 17% of occasions asked, but the health conditions were generally mild childhood afflictions. There was almost no malaria. The more serious AEs provided a clearer picture. The existence of an AE was strongly associated with high hepcidin, as were the AE categories respiratory infections and fever [the latter not surprisingly as this correlates with elevated interleukin-6 (IL6), which is a key up-regulator of hepcidin (*8*)]. Diarrhea and skin infections were not associated with raised hepcidin. Our data additionally indicate that the very high hepcidins in some children cannot be ascribed to any clinical infections per se.

The strong association between hepcidin and granulocyte counts is noteworthy. When expressed as the granulocyte-to-lymphocyte ratio, granulocytes were very strongly positively associated with hepcidin. Environmental enteric dysfunction (EED), characterized by chronic damage and inflammatory infiltrate into the gut mucosa, is widespread in low-income settings and is assumed to be a major driver of growth failure (*20*). Reducing EED is a key intermediary goal of ongoing and recently completed WASH interventions (*21*, *22*). On the basis of the lactulose/mannitol permeability and similar sugar permeability tests, we have previously shown that EED is almost universal in rural Gambian children [e.g., (*23*)]. Given its almost universal prevalence, it would be surprising if EED yielded the very wide range of inflammation and hepcidin levels we report here; additionally, we found that neither fecal calprotectin nor diarrhea was associated with elevated hepcidin. This result replicates the findings of Jaeggi *et al*. (*10*) who, as in this study, found that systemic inflammation was very strongly associated with hepcidin, but found no association of hepcidin with fecal calprotectin or the gut-associated cytokines IL-12 and IL-17. The strong association with respiratory infections therefore stands out as the key finding.

**Fig. 3:**
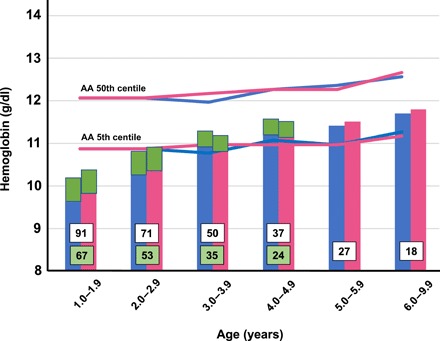
Hb levels in rural Gambian children compared to U.S. reference data for African Americans. Mean Hb levels from healthy rural Gambian children participating in the Keneba Biobank compared to reference data from African Americans (AA) living in the United States (*24*). Blue, boys; pink, girls. 1 to 1.99 years, *n* = 344; 2 to 2.99 years, *n* = 563; 3 to 3.99 years, *n* = 518; 4 to 4.99 years, *n* = 495; 5 to 5.99 years, *n* = 446; 6 to 9.99 years, *n* = 1877. Numbers within columns are percent anemia against WHO cutoff (11 g/dl). Green bars represent WHO meta-analyzed average benefit of iron-plus-multiple-micronutrient randomized trials (*3*, *4*). Green numbers in columns are the predicted anemia rates that would remain after iron-containing micronutrient interventions (*3*, *4*).

The public health challenge of anemia is vividly illustrated in Fig. 3 using data from our Keneba Biobank. The mean Hb level in children aged 1 to 2 years is 1 g/dl lower than the 5th centile for African-American children living in the United States (*24*), and 91% are classified as anemic according to the World Health Organization (WHO) definition. The green bars represent the WHO meta-analyzed average response to iron-plus-multiple-micronutrient interventions conducted under the optimized conditions of randomized controlled trials (*3*, *4*). Applying this benefit would still leave the rural Gambian children well below the African-American 5th centile, and an estimated 67% would still be anemic. Against this background, our current data may have important policy implications. The results suggest that the focus on nutrition-specific interventions (dietary iron sources and anti-nutrients) and specific infections (helminths and malaria) must be complemented by other approaches (*25*, *26*), and they highlight the critical importance of low-grade inflammation. In actuality, half of the children in our study were physiologically blocking iron absorption by a finely evolved hepcidin-dependent mechanism that reduces their risk of infection. These new insights may help explain why iron supplementation programs that have used unphysiological large bolus doses of highly absorbable iron, in an attempt to overcome this blockade, have frequently caused iatrogenic harm (*27*).

The chief strengths of this study are the repeated measures of hepcidin and very comprehensive panels of iron and hematological status and of inflammation in a large sample of children in a typical rural African setting with a very high burden of anemia. A weakness is that we did not measure iron absorption directly and have separated iron absorbers from nonabsorbers using a previously defined threshold established in children of a similar age and in the same setting.

Identifying the key drivers of the inflammation now becomes a research priority. If it derives from a generalized exposure of all mucosal surfaces to pathogens, then the fact that such mild inflammation up-regulates hepcidin suggests that there will be a very high hygiene threshold that needs to be exceeded. However, our data also point to a hitherto undiscovered etiological factor in iron-refractory IDA, namely, respiratory infections, which may, in turn, relate to air pollution such as from wood fires or, in The Gambian setting, dust in the dry season.

Proof-of-principle studies are required to test whether anti-inflammatory interventions could ameliorate IDA, and administration of galacto-oligosaccharides has already shown promise (*28*, *29*). The use of orally active anti-hepcidin compounds that are currently under development could also be informative, but using compounds that overrule the evolved iron-restricting function of hepcidin would need to be conducted under the most rigorous medical cover and is unlikely ever to represent realistic therapeutic options in low-income settings.

We conclude that, in areas with high levels of infections, attempts to combat ID by traditional means will continue to have low efficacy and high levels of side effects unless accompanied by radical improvements in children’s living conditions sufficient to reduce or eliminate even low-grade inflammation with a special focus on ameliorating respiratory infections. An additional inference of relevance to global food policy is that children may be able to maintain adequate iron status even on diets containing low levels of animal products if and when their low-grade inflammation can be eliminated.

## MATERIALS AND METHODS

### Ethics

The study was approved by The Gambia Government/MRC Joint Ethics Committee (SCC/EC ref. no. 1358), and parents gave written consent following a detailed oral description and receipt of a written informed consent document.

### Participants and setting

In the course of a randomized controlled trial to test the utility of a hepcidin-guided screen-and-treat approach to iron supplementation (ISRCTN 07210906) (*13*), we conducted a post hoc exploratory analysis of serial finger-prick plasma hepcidin weekly for 12 weeks (maximum, 13 measurements; total *n* = 5113) in 407 rural Gambian children aged 6 to 23 months at recruitment (see Table 1 for baseline characteristics). The children were recruited in five cohorts between 26 May 2014 and 10 August 2015 to cover both rainy and dry seasons. All children were preselected as having Hb <11 g/dl at screening. At recruitment, 783 families consented to be screened, but 104 could not be called because they had traveled and a further 56 were called but did not attend. Of the 623 who attended, 10 were excluded on health grounds and 64 were below the preset exclusion criterion of <–3 SD for weight-for-age or height-for-age. Venous bleeding was not achieved for four children, one family refused, and one child was found to be too young. Of the remaining 543 children, 136 had Hb values >11 g/dl, leaving 407 children (75%) who were randomized. Randomized children received multiple micronutrient powders (MMPs) either with or without supplemental iron at varying levels: group 1, “universal iron supplementation” with 12 mg of iron (as ferrous fumarate) daily; group 2, weekly “screen-and-treat” iron supplementation using MMP with 12 mg of iron daily for a week if the previous hepcidin was <5.5 ng/ml (otherwise MMP without iron); group 3, weekly “screen-and-treat” iron supplementation using MMP with 6 mg of iron daily for a week if the previous hepcidin was <5.5 ng/ml (otherwise MMP without iron) (*13*). Height and weight were assessed using standard techniques and converted to *z* scores using WHO Anthrops (*30*).

### Hematology and biochemistry

At 0, 49, and 84 days, we took venous blood to perform a full blood count (Medonic M^20M^ GP analyzer) and analyzed plasma iron status (serum iron, UIBC, ferritin, transferrin, sTfR, and TSAT) as well as markers of inflammation (CRP and AGP) (all by Roche COBAS INTEGRA 400 plus). Hepcidin was analyzed using the Bachem Human Hepcidin 25 ELISA (Peninsular Laboratories LLC, San Carlos, USA), which had been validated in a worldwide hepcidin assay harmonization exercise involving both mass spectrometric and immunochemical assays (*31*). The intra-assay coefficient of variation was 8.7%. In 11.2% of cases, where hepcidin levels exceeded the linear portion of the ELISA calibration plot (>50 ng/ml), we repeated the analysis after further sample dilution where the sample was available, but in 7.9% of cases allocated the value of 50 ng/ml. The lower limit of detection was 0.098 ng/ml, and we set half of the detection limit as the lowest possible value (i.e., 0.049 ng/ml).

### Statistical analysis

The power calculations used to determine the sample size were based on the objectives of the clinical trial and have been reported elsewhere (*13*). All participants were included in the current analysis. Variables with skewed distribution (hepcidin, ferritin, CRP, AGP, and fecal calprotectin) were log-transformed. Multilevel linear modeling and linear regression were performed using DataDesk 7.0.2 (Data Description Inc.). All multilevel models included subject (discrete variable and random intercept) and day of study (continuous) as independent variables. Forward selection (acceptance threshold at *P* < 0.05) was used to determine the variables for inclusion in multivariate analysis of factors predicting hepcidin. None of age, sex, length-for-age, or weight-for-age were significant predictors of hepcidin and hence were not included in the model. The relative strengths of associations between hepcidin and its putative regulators were assessed by the *F* ratio. Children’s mean hepcidin values adjusted for iron status (UIBC), inflammation (CRP), or both were computed using the estimated cell means command in the linear models package of DataDesk. On the basis of previous stable isotope iron absorption studies in children within the same setting, we used a hepcidin threshold of 5.5 ng/ml to separate iron absorbers from nonabsorbers (*12*, *16*). Note that the Bachem ELISA test used here is the same as that used to determine the absorption threshold (5.5 ng/ml) (*16*), and longitudinal stability was checked as part of the regular quality control. Children were scored as being infected (yes/no) at the twice weekly fieldworker assessments if the mothers reported any of fever, vomiting, diarrhea, cough, or “other illness” or if the child had an axillary temperature of >37.5°C. Nurse-ascribed AEs were listed as all events or subclassified as fever, diarrhea, respiratory or skin infections, where such events could coexist and were counted separately (e.g., a child with fever and respiratory infection would contribute to each analysis). Malaria contributed only three AEs and hence could not be analyzed.
